# Comprehensive clinical phenotype, genotype and therapy in Yao syndrome

**DOI:** 10.3389/fimmu.2024.1458118

**Published:** 2024-09-20

**Authors:** Hafsa Nomani, Song Wu, Ashmia Saif, Frank Hwang, Jane Metzger, Brianne Navetta-Modrov, Peter D. Gorevic, Ivona Aksentijevich, Qingping Yao

**Affiliations:** ^1^ Division of Rheumatology, Allergy and Immunology, Stony Brook University Renaissance School of Medicine, Stony Brook, NY, United States; ^2^ Applied Mathematics and Statistics, Stony Brook University, Stony Brook, NY, United States; ^3^ Inflammatory Disease Section, National Human Genome Research Institute, National Institutes of Health, Bethesda, MD, United States

**Keywords:** autoinflammatory disease, Blau syndrome, phenotype, genetically transitional disease, genotype, inflammatory bowel disease, NOD2, Yao syndrome

## Abstract

**Objective:**

Yao syndrome (YAOS) is formerly called nucleotide-binding oligomerization domain containing 2 (*NOD2)*-associated autoinflammatory disease.We report a large cohort of YAOS.

**Methods:**

We conducted a retrospective analysis of a cohort of adult patients with systemic autoinflammatory diseases (SAIDs). All patients underwent testing for a periodic fever syndrome gene panel.

**Results:**

A total of 194 patients carried *NOD2* variants, 152 patients were diagnosed with YAOS, and 42 had mixed autoinflammatory diseases with combined variants in *NOD2* and other SAID-associated genes. Demographic, clinical and molecular data were summaried. In sub-group analysis of the 194 patients, individual patients were often identified to carry two or more variants that usually included IVS8 + 158/R702W, IVS8 + 158/L1007fs, IVS8 + 158/V955I, IVS8 + 158/other, or *NOD2/*variants in other SAID genes. Ninety-nine patients carried single variants. Taken together, these variants contribute to the disease in combination or individually.

**Conclusion:**

This largest cohort has provided comprehensive clinical and genotyping data in YAOS. Variants in the *NOD2* gene can give rise to a spectrum from inflammatory bowel disease to autoinflammatory disease.This report further raises awareness of the underdiagnosed disease in the medical community.

## Introduction

Systemic autoinflammatory diseases (SAIDs) primarily derive from abnormal innate immune responses (1). Nucleotide-binding oligomerization domain (NOD)-like receptors (NLRs) are intracellular sensors to pathogen-associated molecular pattern molecules (PAMPs) and play a critical role in innate immune responses (2). Genetic variations in NLRs have been associated with a number of SAIDs. One example is nucleotide-binding oligomerization domain containing 2 (NOD2), a cytosolic sensor to peptidoglycans or muramyl dipeptide (MDP) from bacterial walls. NOD2 contributes to the defense against microorganisms and the regulation of inflammatory processes, primarily in the gut (3). *NOD2* genetic variations are associated with diseases such as Crohn’s disease (CD, OMIM 266600), Blau syndrome (BS, OMIM 186580), and Yao syndrome (YAOS, OMIM 617321) (4).

YAOS is formerly designated as *NOD2*-associated autoinflammatory disease and phenotypically characterized by recurrent fever, dermatitis, arthralgias, gastrointestinal and sicca-like symptoms, distal leg and eyelid swelling (5). Since our initial report in 2011, this disease has been increasingly recognized and reported in North America, Europe, and Asia (6–10). The disease appears more common than initially thought, particularly in the adult patient population. Patients often went through a long diagnostic odyssey before the final disease diagnosis, and approximately 15% of patients filed for disability due to physical and vocational impairments, resulting in a significant burden of disease (5). Due to its association with specific *NOD2* gene mutations, molecular testing is necessary for diagnosis. Because of overlapping clinical manifestations between YAOS and other SAIDs, such as recurrent fevers, an autoinflammatory disease gene panel is usually performed using Next Generation and targeted DNA sequencing for a differential diagnosis. Over the last decade, the clinical phenotype and genotype of YAOS have expanded rapidly (11). Herein, we report the largest single-site cohort of YAOS patients and provide an update on clinical phenotypes and related aspects.

## Patients and methods

### Patient cohort

We have established a large cohort of adult patients with SAIDs at the Center of Autoinflammatory Diseases in Stony Brook University Hospital since February 2016. These patients were referred from across the U.S. and managed by subspecialists in our group, with clinical, biochemical, and genetic data entered into Electronic Medical Records (EMRs). Specifically, the EMRs of SAID patients with *NOD2* mutations were reviewed. Systemic autoimmune diseases, vasculitis, inflammatory bowel disease (IBD), infectious and malignant diseases were excluded after detailed evaluations. All patients underwent molecular testing for an autoinflammatory disease gene panel, including *MEFV, TNFRSF1A, NLRP3, MVK, NLRP12*, and *NOD2*. Approximately 20% of patients also had larger scale genetic testing for an autoimmune and autoinflammatory gene panel at the Molecular Diagnostics Laboratory at Invitae in California. According to our published criteria, YAOS was diagnosed based on characteristic clinical phenotype and a specific *NOD2* genotype, with the exclusion of related diseases (5). Patients with *NOD2* and other variants in two or more different SAID genes were also included to delineate the *NOD2* genetic landscape.

### Statistical analysis

Descriptive statistics was used for analysis. Median+/-IQR were reported for continuous variables; category percentages were reported for categorical variables. All analyses were performed using SAS 9.4 (SAS Institute Inc., Cary, NC).

## Results

### Demographics and clinical phenotype of the largest cohort of YAOS patients

**Figure 1:**
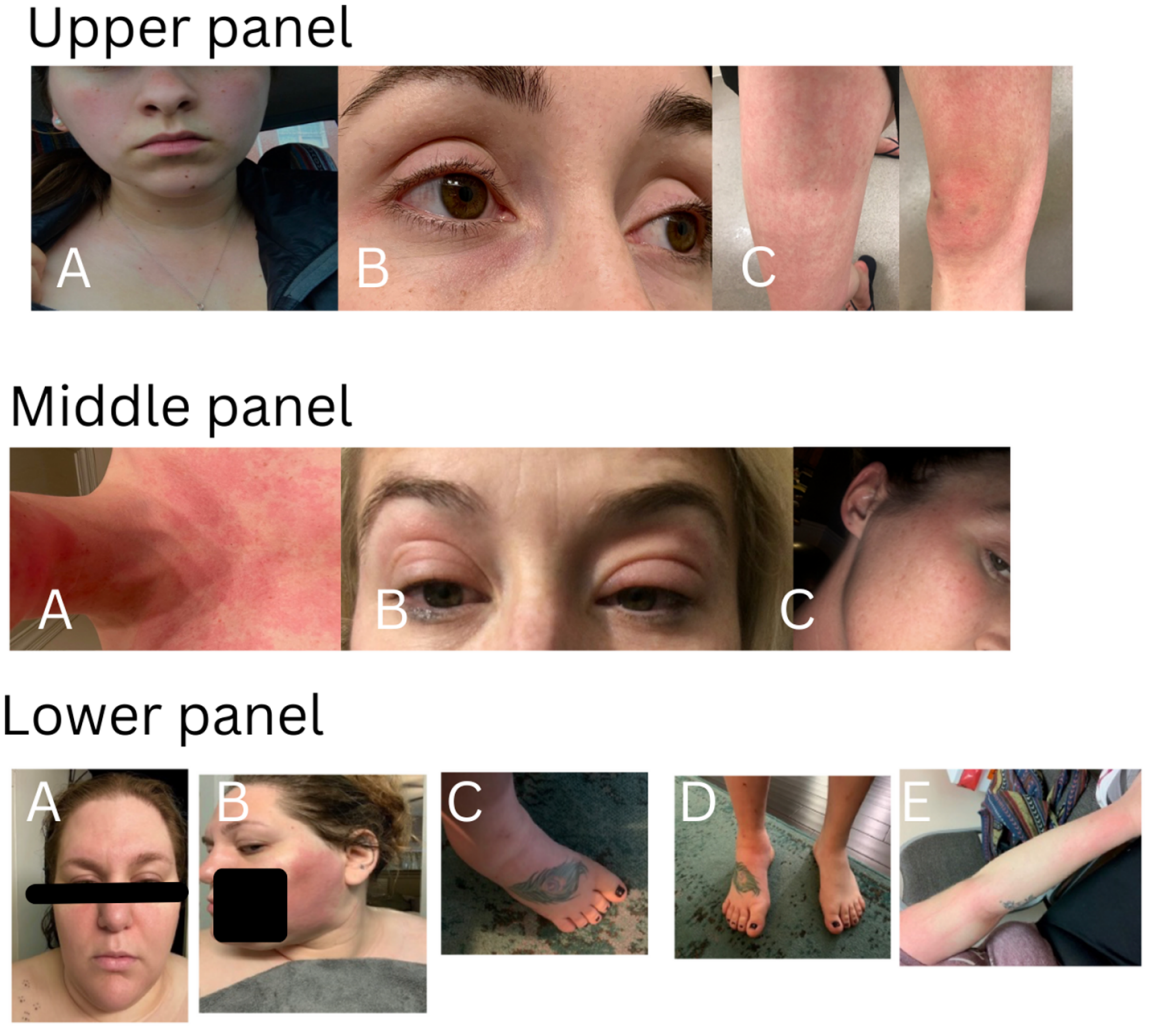
Cutaneous presentations in three representative patients. Upper panel: patchy erythema on cheeks, anterior neck and upper chest **(A)**, eyelid redness and mild puffiness **(B)**, erythematous rash on the thigh and knee with swelling **(C)**. This patient carries heterozygous *NOD2* IVS8 + 158 only and had a good response to IL-1 inhibitor therapy. Middle panel: diffuse redness on the chin, anterior neck and chest **(A)**, bilateral eyelid swelling with discoloration **(B)**, redness on the right upper cheek, temple and external ear **(C)**. This patient carries heterozygous *NOD2* V955I only and had a good response to IL-1 inhibitor therapy. Lower panel: erythematous patches on the face, forehead and bilateral eyelid swelling with discoloration**(A)**, large patchy erythema on the left cheek **(B)**, swollen ankle and foot **(C, D)**, erythematous patches on the arm **(E)**. This patient carries heterozygous and compound *NOD2* IVS8 + 158/1007fs/V955I and had a good response to IL-1 inhibitor therapy.

There were 194 patients in this study cohort, including 152 patients carrying genetic variants only in *NOD2*, and 42 patients with combined genetic variants in *NOD2* and other SAID genes. The demographic data and clinical manifestations and their frequencies from the 152 patients with *NOD2* variants alone are summarized in **Table 1**. The clinical features of the disease were graphically featured previously (5, 12) and are exemplified with three additional representative cases in **Figure 1**.

**Table 1:** Demographic, clinical and laboratory data in YAOS patients.

Variable	Level	Total (N=152)	Variable	Level	Total (N=152)
Age at diagnosis (year)		39 ± 23	Disease duration at diagnosis (year)		8 ± 13
Race	Asian	1 (1%)	Sex	Female	121 (80%)
	Caucasian	150 (99%)		Male	31 (20%)
	Other	1 (1%)	Night sweats	Yes	38 (25%)
Fatigue	Yes	144 (95%)	Fever	Yes	105 (69%)
Headaches	Yes	85 (56%)	Arthralgias	Yes	135 (89%)
Skin Rash	Yes	140 (92%)	Myalgia	Yes	71 (47%)
Lower extremity swelling	Yes	83 (55%)	Gastrointestinal symptoms	Yes	121 (80%)
Oral ulcer	Yes	73 (48%)	Diarrhea	Yes	91 (60%)
Abdominal pain	Yes	106 (70%)	Eyelid swelling	Yes	69 (45%)
Dry eyes and mouth	Yes	90 (59%)	Chest pain	Yes	44 (29%)
Hearing loss/decrease	Yes	14 (9%)	Pericarditis	Yes	19 (13%)
Pleuritis	Yes	16 (11%)	Proteinuria/hematuria	Yes	9 (6%)
Asthma	Yes	35 (23%)	Drug allergy	Yes	90/124 (73%)
Raised ESR/CRP/ferritin	Yes	75 (49%)	Evaluations for allergies	Yes	48/124 (39%)
Food allergy/intolerance	Yes	27/124 (22%)	Hypogamaglobulinemia	Yes	18/40 (45%)

Since YAOS has been previously described in detail, herein we update and focus on certain clinical manifestations that were not previously reported or highlighted. Recurrent fever is common and can last a few hours to weeks. Dermatitis is known to be a common feature primarily manifesting as patchy erythema on the face, chest, or limbs. In addition, urticaria and livedo reticularis were seen in a minority of patients. Eczema-like patches can occur near the earlobe and adjacent areas. Individual patients may present with mixed cutaneous findings of the rashes described above. Generally, rashes are more related to heat than cold and more often occur in summertime or after having a hot shower. Histopathological findings vary, but spongiotic dermatitis and perivascular lymphocytic infiltrate are most common (6). Patients may present with brief episodes of red external ears without pain or thickening distinct from the cartilaginous involvement of relapsing polychondritis. Sicca symptoms are common without typical findings of primary Sjögren syndrome. Eyelid swelling with or without discoloration can occur often in the morning and can be unilateral or bilateral. Patients may have respiratory symptoms, such as chronic sinusitis, asthma-like symptoms, or intermittent episodes of unexplained shortness of breath. Chest pain, pericarditis and pleuritis occurred in 29%, 13%, and 11% of patients, respectively. Several patients developed pleuritis or pericarditis so severe that decortication surgery or placement of a pericardial window was required. Several patients had abnormal cardiac findings including tachycardia and ventricular hypertrophy, dilation, and mild pulmonary hypertension. Gastrointestinal symptoms often include cyclic abdominal pain, bloating and/or nonbloody diarrhea in the majority, whereas other patients may have intermittent episodes of nausea, vomiting or constipation. Extensive gastrointestinal workups did not show convincing evidence of IBD. Oligo- or polyarthralgias are common and usually intermittent without causing articular deformities or destruction. Distal leg swelling of unclear cause, which often involves the ankle and foot, is a characteristic finding, and may be unilateral or bilateral, descriptively resembling lymphedema in some patients. Patients (approximately 16%) may present with episodic superficial (cervical, axillary, or inguinal) and/or deep (intra-abdominal) lymphadenopathy, reactive hyperplasia without granulomatous change mostly. Neurologically, complaints of headaches are common, and several patients were diagnosed with meningitis or ventriculitis. Patient-reported drug allergies and food allergy/intolerance were 73% and 22%, respectively in YAOS (**Table 1**), including cutaneous flushing or allergic phenomena, and even anaphylactic reactions. Forty-one percent of patients underwent evaluations for atopic disorders by allergists/immunologists, and idiopathic chronic urticaria, or mast cell activation syndrome was often suspected. Hypogammaglobinemia can also occur (13). Among the 152 patients with *NOD2* variants only in the current study, 40 were tested for Immunoglobulin (Ig) quantitation, and 45% (18/40) had low Ig of different degrees.

Mild leukocytosis and anemia could occur. Approximately 50% of patients had documented elevated acute phase reactants such as ESR and/or CRP. Patients may have detectable antinuclear antibodies (ANAs) of low titers, but serologic testing for systemic autoimmune diseases is negative for anti-extractable nuclear antigen (ENA), anti-double stranded DNA (dsDNA) antibodies, Rheumatoid factor and Cyclic citrullinated peptide (CCP) antibodies among others.

In summary, YAOS is a systemic inflammatory disease, primarily affecting cutaneous, musculoskeletal, lymphoreticular, cardiopulmonary and gastrointestinal system with rare involvement of internal solid organs. The characteristic clinical phenotypes are recurrent fever, dermatitis, GI symptoms, eyelid swelling, and distal leg swelling to constitute a constellation with other uncharacteristic symptoms. With these in mind, a proper diagnostic workup for autoinflammatory process may be initiated, including genetic testing.

### Genotyping architecture of *NOD2* in YAOS

**Figure 2:**
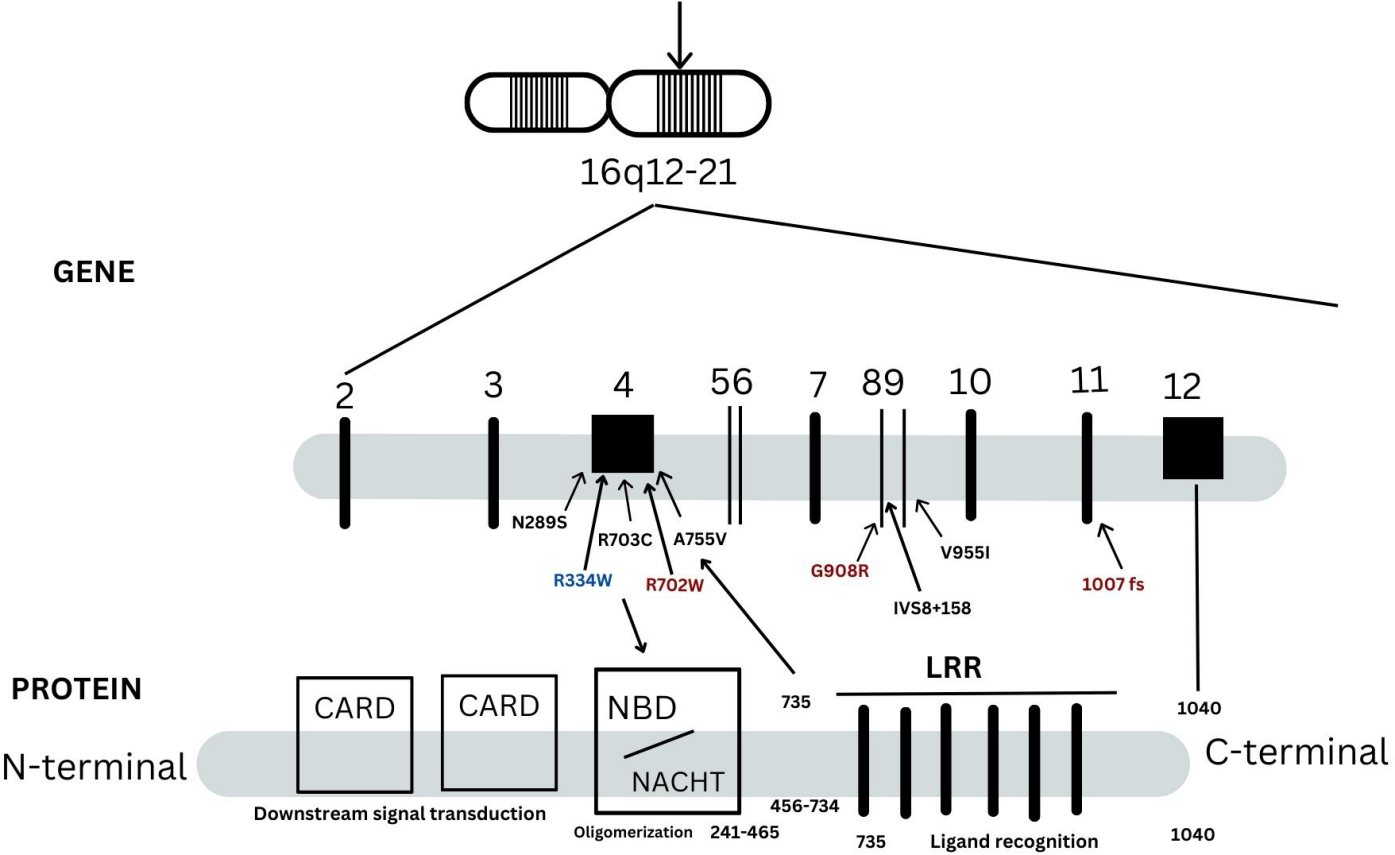
A schematic representation of *NOD2* gene and protein. There are 12 exons in the *NOD2* gene as indicated in vertical lines. NOD2 protein is composed of 1040 amino acids and is divided into three regions: the leucin-rich repeats (LRRs), nucleotide-binding domain (NBD) and caspase recruitment domains (CARDs). They are responsible for bacterial recognition, NOD2 self-oligomerization and down steam signal transduction, respectively. Three main variants in red, 1007fs, Q908R, R702W, are identified in -40% patients with Crohn’s disease. These three NOD2 variants, IVS8 + 158, V955I or other variants are linked to Yao syndrome (YAOS). Most YAOS-associated variants are within the region encoding LRRs and are detected often in combination in individual patients. In a minority of YAOS patients, variants in exon 4(non-Blau syndrome associated variants) and between the exon 4 and the region encoding the LRRs are identified; these are usually rare variants and were detected singly or in combination with the LRR variants. Blau syndrome-associated variants are within exon 4 and are of high penetrance.

**Figure 3:**
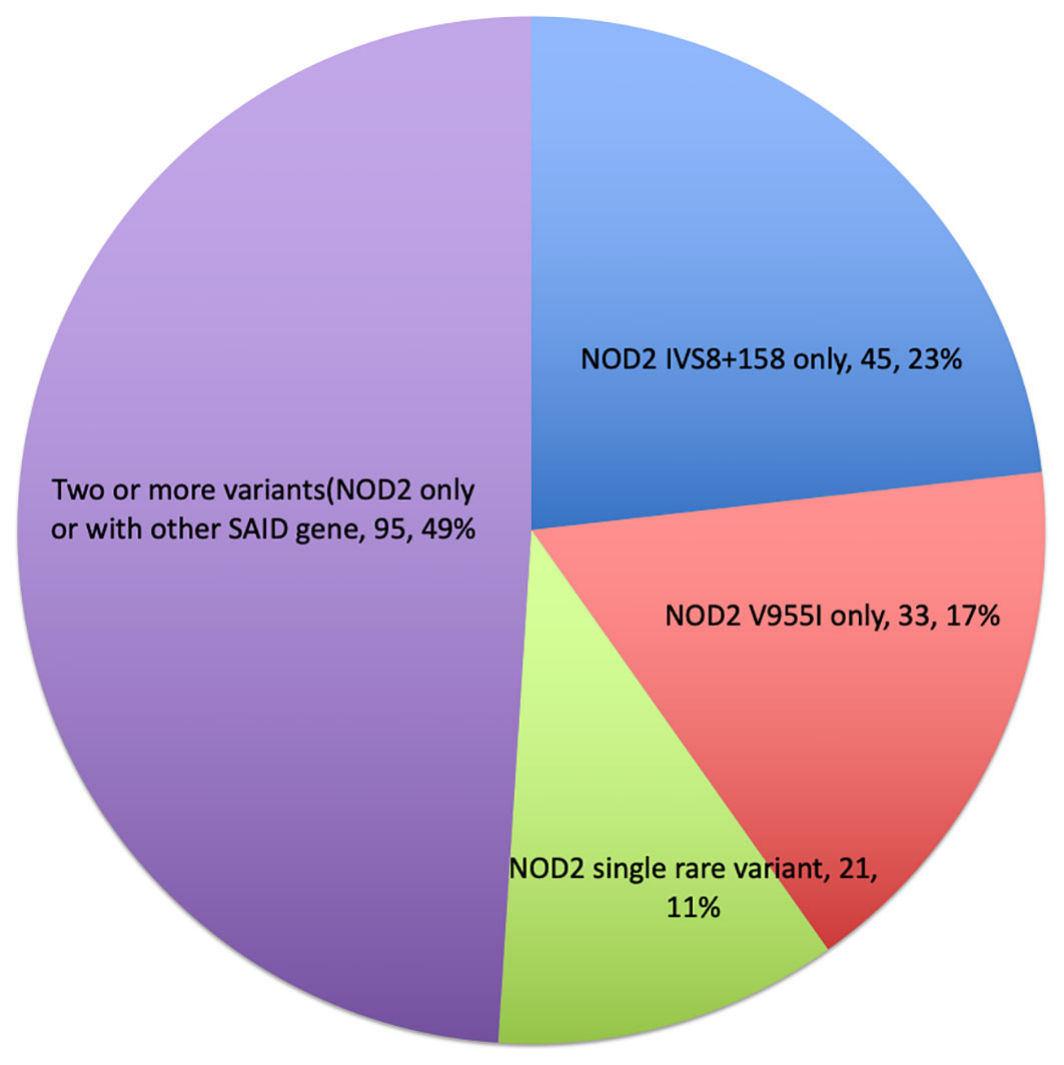
Genotyping distribution in disease (piechart).

A mutational spectrum of *NOD2* has been identified to increase susceptibility to the disease, including 24 gene variants (**Table 2**). Most patients carry variants within the Leucine Rich Repeat (LRR) and adjacent region, and some variants reside in the central NACHT domain encoded by exon 4 (R702W, R703C) (**Figure 2**). Of the 24 variants, 19 are rare (MAF<1%) and 5 are classified as low-frequency or common. Most variants have been previously reported to increase susceptibility to YAOS (11, 12) (7). For example, *NOD2* IVS8 + 158, V955I, R702W, and 1007fs are main variants in YAOS. In our sub-group analysis of the 194 patients with positive *NOD2* variants (**Figure 3**), 95 (49%) patients carried two or more variants that usually included IVS8 + 158/R702W, IVS8 + 158/L1007fs, IVS8 + 158/V955I, IVS8 + 158/N852S, or IVS8 + 158/other (approximately 27%), or *NOD2* with other SAID gene variants (22%). These combinations often consisted of common and low frequency *NOD2* variants. Twenty-three percent of patients carried *NOD2* IVS8 + 158 only, and 17.0% carried V955I only. Thirty-seven (19%) carried 19 rare *NOD2* variants, including 6 single variants and 13 in combination with other variants. Of the 37 patients, 21 carried single rare *NOD2* variants and 16 also carried another *NOD2* or other SAID gene variants. The variant combinations were usually low-penetrance/rare variants in *NOD2* or other SAID genes such as *MEFV*: E148Q or *NLRP3*: Q705K. (**Figure 3**). Genotyping combinations in YAOS can be common and low frequency *NOD2* variants or low frequency or common *NOD2* variants with rare *NOD2* variants. Taken together, these data underpin the contributions of these *NOD2* variants to the disease in combination or individually.

**Table 2:** Genotyping results in Yao syndrome.

NOD2 variant	Exon	Genomic coordinate	Patients (N=194)
IVS8 + 158	Intron 8	16-50756774-C-T	116
V955I	9	16-50757276-G-A	50
R702W	4	16-50745926-C-T	38
L1007fs	11	16-50763778-G-GC	21
G908R	8	16-50756540-G-C	11
R703C	4	16-50745929-C-T	10
A755V	4	16-50746086-C-T	4
N852S#(w/IVS8 + 158, n=3)	6	16-50750810-A-G	3
N289S#(w/IVS8 + 158 and V955I, n=1)	4	16-50744688-A-G	2
D154N#(w/MEFV K695R)	2	16-50733785-G-A	1
T189M	3	16-50741791-C-T	1
R373C	4	16-50744939-C-T	1
S431L#(w/V793M+TNFRSF1A R92Q)	4	16-50745114-C-T	1
R684Q#(w/G908R)	4	16-50745873-G-A	1
R684W#(w/NLRP3 Q705K)	4	16-50745872-C-T	1
A725G#(w/MEFV E148Q)	4	16-50745996-C-G	1
E729D#(w/IVS8 + 158)	4	16-50746009-G-T	1
R744W	4	16-50746052-C-T	1
R791Q#(w/IVS8 + 158 + MEFV 148Q)	4	16-50746194-G-A	1
V793M	4	16-50746200-T-C	1
R830Q#(w/V955I)	5	16-50750524-G-A	1
E843K#(w/IVS8 + 158 + V955I)	5	16-50750562-G-A	2
A918D	8	16-50756571-C-A	2
D925G#(w/IVS8 + 158, n=1)	8	16-50756592-A-G	2

# Variants in parenthesis are added to explain the combinations.

## Discussion

YAOS is an increasingly recognized autoinflammatory disease associated with *NOD2* mutations. In this largest patient cohort, we delineated the genotyping architecture of *NOD2* and updated clinical phenotypes (**Table 1**, **Figure 2**). This study provides a more detailed genetic map and clinical data useful for prompt recognition and diagnosis.

### *NOD2* genotype contributes to different phenotypes

First, the intronic variant *NOD2* IVS8 + 158 (rs5743289) has been identified in approximately 60% of our adult patients, of whom 94% were heterozygote carriers. However, a significant portion of these patients also carrried concurrent one or two low- frequency or rare *NOD2* variants in the form of compound heterozygous variants. For example, *NOD2* IVS8 + 158 can coincide with R702W (missense mutation), 1007fs (frameshift mutation), V955I (missense mutation), N852S(missense mutation), or other *NOD2* variants. In the current study, 26% (50) of patients carried heterozygous *NOD2* V955I, of whom 33 had V955I only and 17 were concurrent with other *NOD2* variants.

Based on our studies and the literature, low-frequency and common variants of *NOD2* can give rise to a spectrum from IBD to SAID. Apart from YAOS, *NOD2* mutations are well known to be associated with CD and BS. Up to 40% of patients with CD carry three main *NOD2* variants, R702W, G908R, 1007fs, located in exons 4, 8 and 11, respectively (14). CD, therefore, is a major differential diagnosis for YAOS given the shared genotypes in both diseases. In a recent study, supervised machine learning was used to classify IBD patients by subtype using whole exome sequecing (WES) data; *NOD2* was found to be the top gene for discriminating CD and ulcerative colitis, regardless of gene panel used (15). In another study of 1,183 pediatric patients with IBD using WES, *NOD2* variants were found to contribute to early-onset CD in which the landscape of *NOD2* variants was defined (16). Compared to the genetic map in the pediatric patients, the *NOD2* genetic architecture as determined by targeted gene panel sequencing in YAOS appears somewhat similar (**Figure 2**). However, biallelic rare and low-frequency *NOD2* variants were identified more often in pediatric patients (8%), either as homozygote or compound heterozygous variants, R702W, 1007fs, and G908R. In our cohort, only one 28-year-old female carried homozygous *NOD2* G908R without IBD. Altogether, these data indicate that homozygotes and compound heterozygotes of these three variants cause early-onset disease, likely due to their double gene dose effect. In addition, BS is an autosomal dominant disease characterized by the triad of granulomatous dermatitis, uveitis and inflammatory arthritis (17). BS primarily occurs in children, and adult-onset disease is extremely rare. Both dermatitis and arthritis are symmetric and persistent, and fever and gastrointestinal symptoms are unusual in the American and European populations (18, 19). In contrast, approximately 50% (26/50) of Japanese patients with BS were reported to have fever, while GI symptoms were nearly absent (20). BS is caused by the high-penetrance *NOD2* variants in exon 4, such as *R334W, R334Q and a dozen other rare NOD2 variants* (20, 21). Of note, these variants were not identified in our current cohort. Taken together, these data support that the *NOD2* genotypes have varying degrees of penetrance and they can contribute to different phenotypes. In fact, the same genotype can be associated with different phenotypes due to contribution of other genetic, developmental, and environmental variables (22, 23).

### Genetic mechanisms underlying YAOS

#### Deciphering disease by genetically transitional disease model

Human genetic disorders have been traditionally classified as monogenic (Mendelian) or complex (polygenic). In Mendelian diseases (Huntington disease), there were slight but no predominant differences in sex ratios (24, 25), as primary mutations are highly penetrant and major determinants in the pathogenesis of diseases. Similarly, a female to male ratio (24/26) was not predominant for BS (20). By contrast, female/male ratio was 52/48 for CD patients without *NOD2* variants and 62/38 for patients with one *NOD2* variant (14). In YAOS, women account for 80% of the patient population. Both CD (up to 40% with *NOD2* variants) and YAOS are associated with *NOD2* variants of incomplete penetrance and only a small proportion of individuals with such variants develop disease. These diseases or disease status cannot be explained by the monogenic disease model. To supplement the traditional dichotomous classification, we recently proposed and defined the Genetically Transitional Disease (GTD) concept (26). GTD refers to a disease status between monogenic and polygenic, where a gene mutation is necessary but not sufficient to cause disease (26). This concept has been now applied to certain rheumatic diseases (27), including autoinflammatory diseases. Human genetic disorders generally result from interactions between candidate genes, genetic background, and environment among others (28). Genetic background refers to all other related genes that may interact with the gene of interest to potentially influence specific phenotype in concert with environment, such as diet (23, 29). GTD highlights the pervasive influence of genetic background together with environment. Unlike monogenic diseases, genetic background (including X chromosome and maternally transmitted mitochondrial DNA) and sex hormones could play more important roles in YAOS and CD with low-penetrance *NOD2* variants, possibly resulting in female predominance (30).

### Two-hit like hypothesis

Notably, in our cohort of patients, approximately 50% of patients carried two or more *NOD2* variants, or *NOD2* with other SAID gene variants, including *MEFV*, *NLRP3* and *NLRP12*. We recently reported that patients with combined *NOD2* and other gene variants may present with mixed autoinflammatory diseases, but YAOS was enriched. Such combinations may contribute to the etiological complexity of disease due to the genetic background in relation to SAID genes (30).

Two-hit hypothesis has been demonstrated in the development of tumors (31). We assumed that a two-hit-like theory, i.e., two or more germline mutational events in the same gene (intragenic) or different genes (extragenic) are required for the development of some SAIDs (27). Approximately 27% patients with YAOS carry two or more low-penetrance *NOD2* variants, while 19% carry one rare *NOD2* variant along with one or more low-penetrance variants in *NOD2* or other SAID genes (**Figure 3**). In CD, *NOD2* gene-dosage effects have been noted. For example, individuals carrying any one of the CD associated risk alleles (R702W, G908R, L1007fs) have two to four fold increased risk for developing CD (32), whereas carriers of two or more of the same *NOD2* variants have a 15–40 fold increased risk for developing CD (14). Individuals with two or more rare and low-frequency *NOD2* variants (homozygotes or compound heterozygotes) have been found to develop early-onset CD (16). Together, these data support our postulate that the two-hit like theory could be operational in the NOD2-associated diseases. We believe that this theory is compatible with intragenic or extragenic epistasis or gene to gene interactions as in our study (30). Based on this theory, some YAOS patients with monoallelic *NOD2* variants may be further examined to search for another one or more germline or somatic mutational events.

### Pathophysiology of NOD2 in NOD2-associated diseases

NOD2 is highly expressed in monocytes, macrophages, dendritic, and paneth cells (3). NOD2 binds MDP and this ligation causes self-oligomerization leading to activation of downstream signal transduction pathways, including NF-kB (33). High-impact BS-associted mutations are gain-of-function and result in constitutive protein activation (34). BS-associated mutations can also result in loss of NOD2 cross-regulatory function (35). In contrast, CD-associated *NOD2* variants are considered loss-of-function with respect to sensing and they impair the NOD2 signaling pathway activation, causing abnormal production of cytokines (36).

*NOD2* IVS8 + 158 is an intronic missense mutation, aka c.2798 + 158 C>T or JW1 (37). Non-coding variants have been increasingly reported to be associated with diseases, and recommendations on clinical interpretations of such variants have been proposed (38). In general, intronic mutations are known to cause disease through alterations in splicing sites, splicing regulatory elements, or disruption of transcription regulatory motifs and non-coding RNA genes (39). Our prior study did not find an alteration in the splicing site of *NOD2* IVS8 + 158, but *NOD2* transcript level and basal p38 mitogen-activated protein kinase (MAPK) activity were significantly elevated in peripheral blood mononuclear cells (PBMCs) from YAOS patients (40). We also demonstrated the presence of dysregulated NOD2 pathway signaling and cytokine profiles in YAOS (40). PBMCs in cocultures with MDP produced higher levels of IL-6 and MAPK in YAOS patients with the *NOD2* IVS8 + 158 variant, suggesting gain-of-function. Cytokines and NF-kB activation were significantly lower, favoring loss-of-function in patients with compound *NOD2* IVS8 + 158/R702W perhaps due to their additive influences, profoundly compromising the NOD2 activation pathway. Using RNA sequencing and transcriptomic analysis, NOD2-mediated signaling pathway was found to be hyperactive with overproduction of pro-inflammatory cytokines such as IL-1 and IL-6 in YAOS patients with NOD2 variant Q902K (41). YAOS is considered to be a GTD and may result from a complex interaction of genetic variants in *NOD2* and genetic background from other innate immune sensor genes, in combination with environmental triggers. For example, gastrointestinal surgeries may trigger or exacerbate disease (5) and we have reported that COVID-19 infection or vaccinations can elicit disease expression or exacerbation (42).

In summary, BS-associated *NOD2* variants are highly penetrant, whereas CD- and YAOS-associated *NOD2* variants are of low penetrance, and both diseases share *NOD2* variants, such as R702W, L1007fs and G908R. Functional study of YAOS has been limited to date. Further study will be needed to refine molecular mechanisms of YAOS.

### Management and prognosis

In the current article, we briefly discuss therapeutic options. Short courses of glucocorticoids are effective for flares and sulfasalazine was effective in 40%. Hydroxychloroquine was tried in many patients with minimal or temporary effects. Methotrexate was ineffective overall. Biologics are second choice if there is failure to improve with or intolerance of sulfasalazine. Unlike BS or CD, IL-1 antagonists are generally effective for YAOS (**Table 3**). In a Greek study of 167 patients with SAIDs, YAOS was diagnosed in 12 patients (7% of the entire cohort), largely substantiating the clinical phenotype, genotype and therapeutic response as we previously reported (10). For non-responders, a Janus kinase (JAK) inhibitor or IL-6 inhibitor may be tried. Based on our experience, sulfasalazine or hydroxychloroquine combined with an IL-1 inhibitor or JAK inhibitor could be used to maximize effectiveness for some cases. While anti-TNFα is a major therapy for BS (43), it produced mixed or temporary improvement for the treatment of YAOS. Further studies are warranted to identify or develop more effective new drugs to treat YAOS.

**Table 3:** Therapeutic data for YAOS patients.

	History of use of HCQ	History of use of MTX	History of use of SSZ	HCQ	MTX	SSZ	IL-1i	TNFi	IL-6i	JAKi	IL-17i	IL-12/23i
**Patients (n)**	13	13	51	8	2	30	57	16	6	4	3	1
**Patients with improved symptoms** **(% Improved)**	Unknown	Unknown	Unknown	5 (62.5%)	0 (0%)	11 (36.7%)	37 (64.9%)	5 (31.3%)	2 (33.3%)	3 (75.0%)	0 (0%)	0 (0%)

HCQ, hydroxychloroquine; MTX, methotrexate; SSZ, sulfasalazine; TNFi, TNF inhibitor; JAKi, JAK inhibitor.

### Genetic counselling

Genetic counseling using the GTD concept may be appropriate and supplemental due to the identification of the *NOD2* variants. This will help patients understand the medical, psychological, and familial implications of genetic contributions to their disease. Approximately 15% of YAOS patients report a positive family history (6). Therefore, we recommend that family members and relatives be genotyped only if they experience similar autoinflammatory symptoms. Prognostically, most patients have recurrent disease flares of varying frequencies, and some may eventually develop chronic and persistent disease. To date, none of our patients have developed CD over a decade long follow up. A minority may develop chronic pain syndrome or fibromyalgia, consequently affecting physical and/or mental function. Some patients in the current study were initially diagnosed with Still’s disease or Periodic Fever, Aphthous Stomatitis, Pharyngitis, Adenitis (PFAPA), in which diagnostic criteria are clinical, as these criteria were established before periodic fever gene testing was available. In those clinical scenarios, we suggest testing for a periodic fever syndrome gene panel, including *NOD2* whole gene sequencing (44). Very rarely were male patients complicated by Whipple’s disease, especially after a long-term immunosuppression (33).

In conclusion, our study of the largest YAOS cohort has updated the clinical phenotype and genotype in the disease. The study provides more comprehensive data and deeper understanding of the contribution of *NOD2* genetic variants to adult-onset inflammatory disease.

### Future perspectives

Given that the Two-hit like theory is postulated to be a potential mechanism of the gene variants in YAOS, functional study of compound *NOD2* variants and combined variants with other related SAID genes might be needed in the future to unveil novel immunobiological mechanisms underlying the disease.

## Data Availability

The original contributions presented in the study are included in the article/supplementary material. Further inquiries can be directed to the corresponding author.
